# Monkeypox virus: A tale of disparity between the wealthy and low-to-middle income nations

**DOI:** 10.1016/j.amsu.2022.104286

**Published:** 2022-07-31

**Authors:** Maha Zahid, Syed Hassan Ahmed, Summaiyya Waseem, Taha Gul Shaikh, Khabab Abbasher Hussien Mohamed Ahmed, Irfan Ullah

**Affiliations:** Dow University of Health Sciences, Karachi, Pakistan; Dow University of Health Sciences, Karachi, Pakistan; Dow University of Health Sciences, Karachi, Pakistan; Dow University of Health Sciences, Karachi, Pakistan; Faculty of Medicine, University of Khartoum, Khartoum, Sudan; Kabir Medical College, Gandhara University, Peshawar, Pakistan; Institute of Public Health and Social Science (IPH&SS), Khyber Medical University, Peshawar, Pakistan

Dear Editor,

In the middle of the COVID-19 pandemic, a rapidly emerging outbreak of a formerly existent yet neglected virus, monkeypox, has alarmed the world. A member of the family Poxviridae and the genus Orthopoxvirus, monkeypox virus (MPXV), is a brick-shaped, enveloped, double-stranded DNA virus closely related to now-extinct smallpox (variola virus) that previously plagued humans [1]. MPXV is classified into two distinct genetic clades, West African and Congo Basin, the latter causing more severe illness with a case fatality rate (CFR) of up to 11% [2].

In 1970, the Democratic Republic of the Congo reported the discovery of the first human MPXV infection, where it became endemic. Ever since then, the virus has spread to other African nations, primarily Central and West Africa [3]. In 2003, for the first time, an outbreak emerged outside of Africa, in the United States of America (USA), secondary to imported infected animals from Ghana [2]. More recently, there has been an atypical emergence throughout the non-endemic, previously disease-free countries, which has caused substantial fear and anxiety among populations. According to the World Health Organization (WHO), a total of 2103 laboratory-confirmed and one reported fatality across 42 nations in five WHO regions have been reported from 1st January to 15th June 2022, with 98% of them being reported since May 2022 [4]. Established patients have no traveling history to endemic regions; therefore, it appears that the outbreak is extremely exceptional [5].

The virus predominantly transmits via close contact with contaminated objects or bodily fluids, such as during sexual activity or respiratory droplets. It can also transmit to health care providers who have had close, sustained contact with a patient [4]. Following exposure, patients often undergo a feverish prodrome lasting 5–13 days and frequently involves lymphadenopathy, malaise, headaches, and muscle aches. The typical deep-seated, vesicular, or pustular skin rash appears 1–4 days later. However, monkeypox cases, connected to the recent outbreak, have started abnormally, with lesions in the vaginal and perianal region but no subjective fever or other prodromal signs [6]. Moreover, infected patients may experience extracutaneous manifestations such as secondary skin or soft-tissue infection (19%), pneumonitis (12%), ocular problems (4–5%), and encephalitis (<1%) [7]. Monkeypox can be diagnosed through laboratory testing using Reverse Transcription, Polymerase Chain Reaction (RT-PCR) [8]. Currently, supportive care marks the mainstay of treatment, while Tecovirimat and Brincidofovir, two FDA-approved antivirals, can also be used [5]. WHO has started the clinical and public health incident response, the goal of which entails identification of cases, tracking down contacts, laboratory analysis, clinical management, isolation, and implementation of infection prevention and control measures. Wherever possible, genomic sequencing of viral DNA is being conducted [4].

Monkeypox has been endemic in the African region for the past five decades. However, the fact that new cases are recently showing up outside Africa highlights the disease's geographical growth potential and its global importance. Owing to the ongoing COVID-19 pandemic, the healthcare resources are strapped, hence the possible pandemic risk carried by the virus should not be underestimated [3]. It is evident that emphasis is solely laid when certain diseases strike high-income nations, and this is a perfect example of how the system has insufficiently failed to address "epidemic preparedness" and "global health". It also exemplifies the value disparity between wealthy nations and the rest of the globe concerning how people's health is dealt with [9].

With the current economic instability and the toll COVID-19 took on healthcare systems, many national health systems cannot afford pharmaceutical tools like vaccinations, diagnostic kits, and antivirals. Therefore, it is imperative to consider the beneficial outcomes of various interventions such as raising awareness, surveillance including self-reporting, and quarantining measures [8]. Although the possibility of occurrence in low-to-middle income (LMI) countries is uncertain, given their current economic crisis secondary to the COVID-19 pandemic, safety measures are essential to prevent the viral spread and greater havoc. Travelers from endemic regions should be screened routinely, while suspected or confirmed cases should also be quarantined for a prodromal period. Hospitals should have well-equipped isolation units on standby to place patients in quarantine. Moreover, as the most vulnerable group, healthcare personnel should be fully equipped with the required Personal Protective Equipment (PPEs), especially in light of the global shortage witnessed during the COVID-19 epidemic. The course of outbreaks is significantly shaped by laboratories, however, several LMI countries such as Pakistan currently lack viral diagnosis facilities [10]. Currently, mass immunization against monkeypox is neither necessary nor advised by WHO [4]. However, vaccine supplies should be stocked for pre and post-exposure prophylaxis in high-risk groups. The vaccine, MVA-BN (modified vaccinia Ankara-Bavarian Nordic) has been licensed for viral prevention in the USA. Lastly, international funding must be made readily available to assist LMI countries cope with the developing situation and establish a globally extensive surveillance system to understand the continually changing epidemiology of this emerging illness [9].

## Financial disclosure

None.

## Provenance and peer review

Externally peer reviewed, not commissioned.

## Annals of medicine and surgery

The following information is required for submission. Please note that failure to respond to these questions/statements will mean your submission will be returned. If you have nothing to declare in any of these categories then this should be stated.

## Conflicts of interest

No conflicts of interest to declare.

## Sources of funding

No funds were granted for this work.

## Ethical approval

Not required.

## Consent

Written informed consent was obtained from the patient to publish this case report and any accompanying images.

## Author contribution

Not applicable.

## Registration of research studies

1. Name of the registry: Not required.
2. Unique Identifying number or registration ID: Not applicable.
3. Hyperlink to your specific registration (must be publicly accessible and will be checked):

## Guarantor

Irfan Ullah.

## Declaration of competing interest

The authors declare no conflict of interest.
